# Global implications of crop‐based bioenergy with carbon capture and storage for terrestrial vertebrate biodiversity

**DOI:** 10.1111/gcbb.12911

**Published:** 2021-12-20

**Authors:** Steef V. Hanssen, Zoran J. N. Steinmann, Vassilis Daioglou, Mirza Čengić, Detlef P. Van Vuuren, Mark A. J. Huijbregts

**Affiliations:** ^1^ Department of Environmental Science Radboud Institute for Biological and Environmental Sciences Radboud University Nijmegen The Netherlands; ^2^ Environmental Systems Analysis Group Wageningen University & Research Wageningen The Netherlands; ^3^ PBL Netherlands Environmental Assessment Agency The Hague The Netherlands; ^4^ Copernicus Institute of Sustainable Development Utrecht University Utrecht The Netherlands

**Keywords:** BECCS, biodiversity, biomass, climate change, land‐use change, species loss, trade‐offs

## Abstract

Bioenergy with carbon capture and storage (BECCS) based on purpose‐grown lignocellulosic crops can provide *negative* CO_2_ emissions to mitigate climate change, but its land requirements present a threat to biodiversity. Here, we analyse the implications of crop‐based BECCS for global terrestrial vertebrate species richness, considering both the land‐use change (LUC) required *for* BECCS and the climate change prevented *by* BECCS. LUC impacts are determined using global‐equivalent, species–area relationship‐based loss factors. We find that sequestering 0.5–5 Gtonne of CO_2_ per year with lignocellulosic crop‐based BECCS would require hundreds of Mha of land, and commit tens of terrestrial vertebrate species to extinction. Species loss per unit of negative emissions decreases with: (i) longer lifetimes of BECCS systems, (ii) less overall deployment of crop‐based BECCS and (iii) optimal land allocation, that is prioritizing locations with the lowest species loss per negative emission potential, rather than minimizing overall land use or prioritizing locations with the lowest biodiversity. The consequences of prevented climate change for biodiversity are based on existing climate response relationships. Our tentative comparison shows that for crop‐based BECCS considered over 30 years, LUC impacts on vertebrate species richness may outweigh the positive effects of prevented climate change. Conversely, for BECCS considered over 80 years, the positive effects of climate change mitigation on biodiversity may outweigh the negative effects of LUC. However, both effects and their interaction are highly uncertain and require further understanding, along with the analysis of additional species groups and biodiversity metrics. We conclude that factoring in biodiversity means lignocellulosic crop‐based BECCS should be used early to achieve the required mitigation over longer time periods, on optimal biomass cultivation locations, and most importantly, as little as possible where conversion of natural land is involved, looking instead to sustainably grown or residual biomass‐based feedstocks and alternative strategies for carbon dioxide removal.

## Graphical abstract

Bioenergy with carbon capture and storage (BECCS) could provide negative CO_2_ emissions to mitigate climate change, but if based on crops, the associated land requirements threaten biodiversity. Here, we show that sequestering 0.5–5 GtCO_2_/year with lignocellulosic crop‐based BECCS requires hundreds of Mha of land and would commit tens of terrestrial vertebrate species to extinction. When considered over a 30‐year evaluation period, these land‐use impacts likely outweigh the benefits of prevented climate change to vertebrate richness, though this could reverse over longer evaluation times. We conclude that species loss is reduced with longer BECCS lifetimes and optimal land allocation.

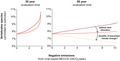

## 1 INTRODUCTION

Most climate change mitigation pathways consistent with 1.5–2°C global warming require negative greenhouse gas (GHG) emissions to offset emissions of hard to abate sectors (e.g. heavy transport and industry) and balance near‐term exceedance of emission budgets (Rogelj et al., 2018). Out of several options to achieve negative emissions (Fuss et al., 2016, 2018; Smith et al., 2016), bioenergy with carbon capture and storage (BECCS) currently features most prominently in mitigation pathways, partly because it is one of the few options that is well represented in the integrated assessment models that underlie mitigation pathways (Rogelj et al., 2018). In the BECCS production chain, atmospheric CO_2_ is taken up by growing biomass, which is then combusted to generate energy, while the released CO_2_ is largely captured and geologically stored, resulting in negative emissions (Azar et al., 2010; Gough & Upham, 2011; Kemper, 2015; Obersteiner et al., 2001). The reasons BECCS can be considered attractive (for instance in integrated assessment models) are that it forms a combination of existing technologies, is scalable, yields useful energy and may have lower costs than other negative emission technologies (Fuss et al., 2016, 2018; Hepburn et al., 2019; Muratori et al., 2020; Smith et al., 2016).

BECCS based on purpose‐grown biomass could (biophysically) contribute to climate change mitigation via negative emissions, depending on cultivation location, treatment of initial vegetation and evaluation period (Hanssen et al., 2020; Harper et al., 2018). This should not be mistaken for a plea that large‐scale biomass cultivation for BECCS is a desirable scenario (Creutzig et al., 2021). It has been well established that dedicated biomass cultivation for BECCS would likely have large environmental impacts through its land requirements and, depending on crop and location, its water and nutrient use (Ai et al., 2021; Bonsch et al., 2016; Fajardy et al., 2018; Heck et al., 2018; Kemper, 2015; Smith et al., 2016; Stoy et al., 2018). In addition, crop cultivation for BECCS could compete for land with food production (Doelman et al., 2018; Fujimori et al., 2019; Hasegawa et al., 2018, 2020). Other biomass feedstocks with lower impacts are therefore more attractive for BECCS, including (i) wastes and residues from agriculture and forestry (Daioglou et al., 2015; Hanssen et al., 2019; Pour et al., 2018), (ii) biomass from sustainably managed forests, for example with selective logging or continuous cover forestry (Dale, Kline, et al., 2015; Goh et al., 2020; Hanssen et al., 2020; Lundmark et al., 2016; Peura et al., 2018) or (iii) cultivated biomass specifically grown on marginal or abandoned agricultural lands (Campbell et al., 2008; Gelfand et al., 2013) using biodiverse, local and high‐yielding mixtures of species (Robertson et al., 2017; Tilman et al., 2006).

Considering, however, that (i) the combined availability of these low‐impact feedstocks for BECCS is uncertain—amplified by the many competing uses for this biomass (e.g. for chemicals and construction materials)—and (ii) many mitigation pathways consistent with 1.5–2°C warming explicitly rely on dedicated bioenergy crops in addition to residues for BECCS‐based negative emissions (Rogelj et al., 2018), the consequences of dedicated biomass production for negative emissions should be very clear. In particular, the land requirements to generate negative emissions warrant additional understanding of the associated costs to biodiversity, which is already in sharp decline from pressures like land conversion, over‐exploitation and climate change—with any further losses widely considered unacceptable (Barnosky et al., 2011; Ceballos et al., 2017; Dirzo et al., 2014; Hoffman et al., 2010; IPBES, 2019).

Previous work concluded that it is impossible to convert additional natural land for BECCS without further transgressing the planetary boundary of biosphere integrity (Heck et al., 2018), which is in line with local assessments that even under optimal management, conversion of natural land remains the primary driver of biodiversity loss (e.g. Williams et al., 2020). This means biomass production for energy should be limited to marginal land and existing production landscapes (Núñez‐Regueiro et al., 2019), though biomass extraction can also reduce biodiversity in managed landscapes (Powell & Lenton, 2013). Heck et al. (2018) based their analysis on the biodiversity intactness index, a local biodiversity indicator representing the relative abundance of native species in an area under anthropogenic use. The *global* relation between negative emissions from bioenergy crop‐based BECCS and global species extinctions is likely to point in the same direction. However, the effect size is currently not well understood.

Paradoxically, while the land conversion for BECCS forms an additional strain on biodiversity, contribution of BECCS to preventing climate change could also prevent biodiversity loss (Thomas et al., 2004; Urban, 2015). Climate‐explicit species distribution modelling has suggested that the impact of bioenergy cropland expansion on global terrestrial vertebrate species richness offsets the positive effects of prevented climate change (Hof et al., 2018), but this did not include negative emissions. A study based on species–area relationship (SAR) modelling has suggested that these offsets remain even when bioenergy is *combined* with carbon capture and storage (Powell & Lenton, 2013).

Here, we explore the relation between negative emissions from lignocellulosic crop‐based BECCS and their impacts on global biodiversity, that is terrestrial vertebrate species becoming committed to global extinction. We combine full life cycle, spatially explicit negative emission potentials for BECCS electricity (based on Hanssen et al., 2020) with global‐equivalent biodiversity loss factors that link local land‐use change (LUC) for BECCS to global vertebrate species richness (Chaudhary & Brooks, 2018). We show the contribution to global‐equivalent species loss of negative emissions across different biomass cultivation locations. Based on these results, we derive global species loss curves at increasing levels of negative emissions from crop‐based BECCS under different land allocation criteria. Our approach is explicitly not a scenario analysis of large‐scale bioenergy cropland expansion, but the analysis does exclude the global food production system to account for indirect LUC, as well as protected natural areas. As a final and preliminary exploration, we compare the biodiversity impact of LUC with the potentially beneficial effect of BECCS‐mitigated climate change. Notably, we include the temporal scope of these analyses by looking at 30‐ and 80‐year evaluation periods for BECCS.

## 2 MATERIALS AND METHODS

### 2.1 Negative GHG emissions from BECCS

Negative GHG emissions from BECCS refer to the *net* amount of CO_2_ that can be taken out of the atmosphere and geologically stored, while considering LUC and supply chain GHG emissions, crop yields, bioenergy conversion efficiencies and carbon capture rates. We derived annual negative emission potentials (tonne CO_2_‐eq./ha/year; Figure S1) for each 0.5° × 0.5° grid cell based on Hanssen et al. (2020), multiplying that study's emission factors for BECCS (tonne CO_2_‐eq./GJ) with bioenergy supply potentials (GJ/ha/year). These emission factors and potentials were based on: (i) spatially explicit (changes in) carbon stocks and bioenergy crop‐specific yield estimates obtained from the LPJml global vegetation and hydrological model (Beringer et al., 2011; Müller et al., 2016) coupled to the IMAGE integrated assessment model (Stehfest et al., 2014), and (ii) literature‐based supply chain emissions, conversion efficiencies and carbon capture rates (for a detailed description, see Hanssen et al., 2020). We specifically used values for electricity with carbon capture and storage (90% capture rate) produced from rainfed lignocellulosic bioenergy crops: either fast‐growing grasses like *Miscanthus* and switchgrass, or short‐rotation coppicing of *Eucalyptus* species, willow or poplar, depending on which results in the greatest amount of net negative emissions for each cultivation location. We assumed that 80% of *stem* biomass present before bioenergy crop plantation establishment is used to produce BECCS electricity and all remaining initial biomass is burned on‐site.

We determined cumulative negative emissions from crop‐based BECCS per grid cell (in tonne CO_2_‐eq.) by multiplying the cell's negative emission potential (tonne CO_2_‐eq./ha/year) with the area in the cell available for BECCS (ha) and the time period considered (years). We investigated the influence of this evaluation period, by considering both a 30‐ and an 80‐year evaluation period. The 30‐year time span reflects typical plantation lifetimes and shows the short to medium‐term climate change mitigation potential (or the potential towards 2100 when starting later in the century); it is also representative of the assumed policy horizon during which BECCS systems may be maintained, after which their continuation might not be guaranteed. The 80‐year evaluation time was considered because it corresponds with the duration of mitigation pathways towards the year 2100 and shows the long‐term potential of BECCS. The evaluation period strongly affects the amount negative emissions achieved for several reasons. Firstly, because initial emissions from LUC have to be compensated by subsequent BECCS‐based carbon sequestration to achieve negative emissions, and the evaluation period effectively sets the amortization period for these initial LUC emissions. Secondly, a longer evaluation period simply leads to more crop rotations and more carbon sequestration. Thirdly, over longer evaluation periods, the longer amortization period means that more locations can yield negative emissions.

### 2.2 Analysed land areas

In this analysis, we considered all land areas that (under cultivation) could result in negative emissions via BECCS, to ultimately estimate for all these areas what their land conversion for BECCS would mean in terms of global biodiversity loss. Grid cells used for food provisioning (cropland and pastures) were excluded from the analysis due to potential indirect LUC effects of their use for bioenergy, as were urban areas. Furthermore, water bodies and natural protected areas were excluded, resulting in the following list of excluded areas:

- Grid cells with very low bioenergy crop yields (i.e. yields below 2.5 tonne wet biomass per hectare per year, which is equivalent to 5% of the global maximum yield based on LPJml).
- Grid cells in which no net negative emissions can be achieved, which differs over the specified evaluation periods and was based on Hanssen et al. (2020).
- Grid cells classified as current or future urban area, cropland or pasture (within the 21st century) according to the SSP2 ‘middle of the road’ baseline scenario in the IMAGE integrated assessment model (Stehfest et al., 2014).
- Water bodies within grid cells.
- (Parts of) grid cells that are currently protected areas (UN WCMC, 2019) and/or ‘intact forests’ (Potapov et al., 2017), which are defined as natural areas (including non‐forest ecosystems) without human activities that are larger than 50 km^2^ and at least 10 km at their broadest point, see also Figure S2.

The remaining areas that *were* included in the analysis thus comprise among others: various (unprotected and non‐intact) natural forests, grasslands and shrublands, secondary forests, abandoned agricultural land and marginal land.

### 2.3 Biodiversity loss from LUC

As global metric of biodiversity loss from LUC, we used the global‐equivalent potential vertebrate species loss factors that have been derived by Chaudhary and Brooks (2018) to determine the influence of LUC on biodiversity loss. Based on SARs, these species loss factors (number of species that become committed to global extinction per ha of land used) have been derived for four classes of terrestrial vertebrates (based on 6251 amphibian, 3384 reptile, 5386 mammal and 10,104 bird species) and for 804 terrestrial ecoregions across the globe (Figure S3). The SARs that these loss factors are based on take into account: the number of original species present in the ecoregion, the loss of natural habitat and the average preference of species for new artificial habitat types. A vulnerability score (based on range sizes and IUCN red list status) is assigned to each species group–ecoregion combination to reflect the vulnerability to extinction on a global scale of the both endemic and non‐endemic species living within that ecoregion.

The species loss factors have been determined for different land‐use types and land‐use intensity levels; we selected intensive plantation forestry to represent the bioenergy crop plantations. In 30 out of 804 ecoregions, no factors for intensive plantation forestry had been derived. In these instances, we used the factors for intensive agriculture (22 ecoregions) or, when these were not unavailable either, clear‐cut forestry (six ecoregions). For two remaining small pacific island ecoregions, no relevant factors were available; these were excluded from the assessment. Ultimately, biodiversity loss from LUC (i.e. contribution to species becoming committed to global extinction) was determined for each vertebrate class and each 0.5° × 0.5° grid cell, by multiplying each cell's species group–ecoregion loss factor with the area of the cell that used for BECCS (see Section 2.1).

### 2.4 Global biodiversity loss curves

After both biodiversity loss from LUC and cumulative negative emissions from BECCS over the considered evaluation periods were quantified per grid cell, their relation at the global scale was derived as a biodiversity loss curve to cumulative carbon sequestration. This was done separately for the 30‐ and 80‐year evaluation periods, which in both cases were also assumed to be the (minimum) lifetimes of bioenergy crop‐based BECCS. In both cases, the relation between biodiversity loss from LUC and negative emissions can have multiple shapes, depending on what locations are used for BECCS. We created biodiversity loss curves for three land allocation criteria:

- Use land with the largest carbon negative emissions potential. Grid cells with the largest cumulative negative emission potential (tonne CO_2_‐eq./ha) are selected first, until all grid cells with net negative emissions are selected. This minimizes land‐use requirements.
- Use land with the lowest biodiversity loss. Grid cells with the lowest biodiversity loss due to land conversion (species/ha; across all four studied taxa) are selected first. This minimizes biodiversity loss per amount of land cultivated.
- Use land with the lowest biodiversity loss per negative emission potential (species/tonne CO_2_‐eq.). This minimizes biodiversity loss per negative emissions achieved.

### 2.5 Prevented biodiversity loss from mitigating climate change

Mitigating climate change could help conserve biodiversity. We therefore contrasted biodiversity loss (i.e. species committed to extinction) due to LUC for BECCS with an estimate of the prevented biodiversity loss of limiting climate change through BECCS. This prevented biodiversity loss was estimated using Equation (1).

(1) $$\text{PBL}=\frac{\text{PBL}}{\Delta T}\cdot \frac{\Delta T}{\text{NE}}\cdot \text{NE,}$$

where PBL is the prevented biodiversity loss (in % of species saved), ∆*T* is the difference in temperature (in °C) and NE are the negative emissions (in teratonne CO_2_ [10^15^ kg]).

The percentage of species saved per °C of warming prevented (PBL/∆*T*) was estimated based on a meta‐regression by Urban (2015) that includes various terrestrial species groups such as vertebrates, plants and insects. Specifically, we looked at temperature change starting from two ‘baseline’ levels of 2.8 and 4.3°C (pre‐industrial) mean global temperature increase (in line with RCP 6 and 8.5; Clarke et al., 2014; Table S1). The effect of negative emissions on global temperature change (∆*T*/NE) was based on the transient climate response to cumulative carbon emission (TCRE) values reported by Van Vuuren et al. (2020).

### 2.6 Estimated uncertainty in the trade‐off between LUC and mitigated climate change

In order to better understand the trade‐off between the negative effects on biodiversity of LUC and the positive effects of preventing climate change, we made a first estimate of the statistical uncertainty of both effects. For LUC, the uncertainty ranges for ecoregion‐specific species richness loss factors were used based on Chaudhary and Brooks (2018). They were considered fully correlated across all ecoregions and specified as 2.5th–97.5th percentile uncertainty ranges.

For mitigated climate change, statistical uncertainty consisted of two components. First, uncertainty in the amount of species saved per °C of warming prevented was based on Urban (2015), using the reported 95% confidence interval (Table S1). This (asymmetric) uncertainty was modelled here using a log‐normal distribution, with percentiles converted to a standard deviation of the log values following Slob (1994). Second, uncertainty in the effect of negative emissions on global temperature was modelled as the normal distribution of TCRE values (0.62 ± 0.12 SD °C/Ttonne CO_2_) reported by Van Vuuren et al. (2020). The overall uncertainty in prevented biodiversity loss per negative emissions was then determined by taking the products of 100,000 random samples of both distributions, for each of the four evaluation‐period and temperature‐scenario combinations, and was reported as the 2.5th–97.5th percentile uncertainty range.

While this approach includes the statistical uncertainty in the individual effects of LUC and prevented climate change, as reported by original authors, it does not cover all aspects of uncertainty. Most importantly, the interaction between LUC and prevented climate change is uncertain but not accounted for in these estimates, as further discussed in Section 4.

## 3 RESULTS

### 3.1 Biodiversity loss from LUC for BECCS‐based negative emissions

**FIGURE 1:**
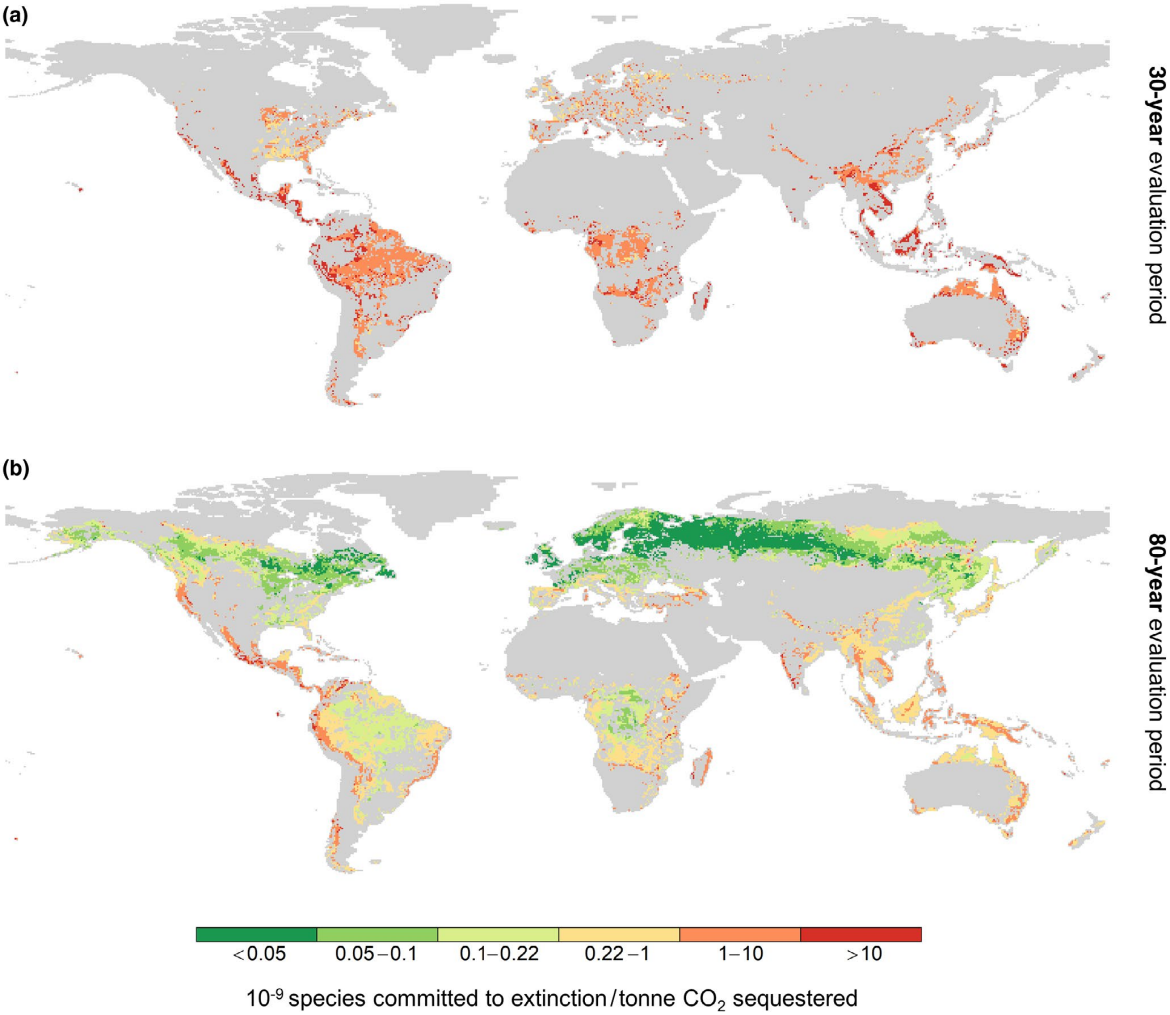
Global‐equivalent biodiversity loss associated with land‐use change for BECCS‐based negative emissions. Indicated are estimates of the potential number of terrestrial vertebrate species committed to extinction due to land use for lignocellulosic crop‐based BECCS, expressed in 10^−9^ species per tonne of CO_2_ sequestered with BECCS, over (a) a 30‐year evaluation period and (b) an 80‐year evaluation period. Biodiversity loss and negative emissions per hectare are also separately presented in Figures S1 and S3 respectively. Grey areas were excluded from our analysis and comprise: agricultural land (cropland and pasture), urban areas, inland waters, protected areas and intact forests, areas with low bioenergy crop yields (<5% of global maximum yields) and areas that do not achieve net CO_2_ sequestration over the time period considered. This means 389 and 241 ecoregions were excluded for the 30‐ and 80‐year evaluation periods respectively. Note that all protected areas and intact forests (Figure S2) are excluded from our analysis, but that values for grid cells that are partly protected areas or intact forests are plotted on these maps. BECCS, bioenergy with carbon capture and storage

Figure 1 shows the estimated potential global biodiversity loss (i.e. vertebrate species committed to global extinction) from LUC for the production of BECCS‐based negative emissions at a given location. Over a 30‐year evaluation period (Figure 1a), cumulative negative emissions are relatively limited, and the biodiversity losses per unit of negative emissions achieved are therefore highest. In almost all locations, sequestering 1 tonne of CO_2_ could contribute the equivalent of 10^−9^ species becoming committed to extinction at the global scale, which over larger areas would translate to one species per Gtonne CO_2_ sequestered. In many tropical regions, however, potential species loss is more than 10 times higher.

An 80‐year evaluation period results in much larger cumulative negative emissions per area converted. So even while biodiversity loss per area converted stays the same, biodiversity loss per tonne of CO_2_ sequestered is lower (Figure 1b). Over this 80‐year period, BECCS could also generate net negative emissions in more locations. Potential global‐equivalent species loss is still very high (1–10+ species/Gtonne) in areas with high (endemic) biodiversity, typically tropical areas, coastal areas and islands, such as in Southeast Asia and Central America.

The geographical patterns of biodiversity loss are similar across the different terrestrial vertebrate classes, except that conversion of cooler areas results in fewer global extinctions of reptile and amphibian species, as fewer of these species are home to these areas (Figure S4). Furthermore, the global patterns of potential LUC‐related biodiversity loss for negative emissions are more strongly influenced by species loss factors, which vary by five orders of magnitude (Figure S3), than by negative emissions potential (Figure S1).

### 3.2 Global biodiversity loss curves for BECCS‐based negative emissions

**FIGURE 2:**
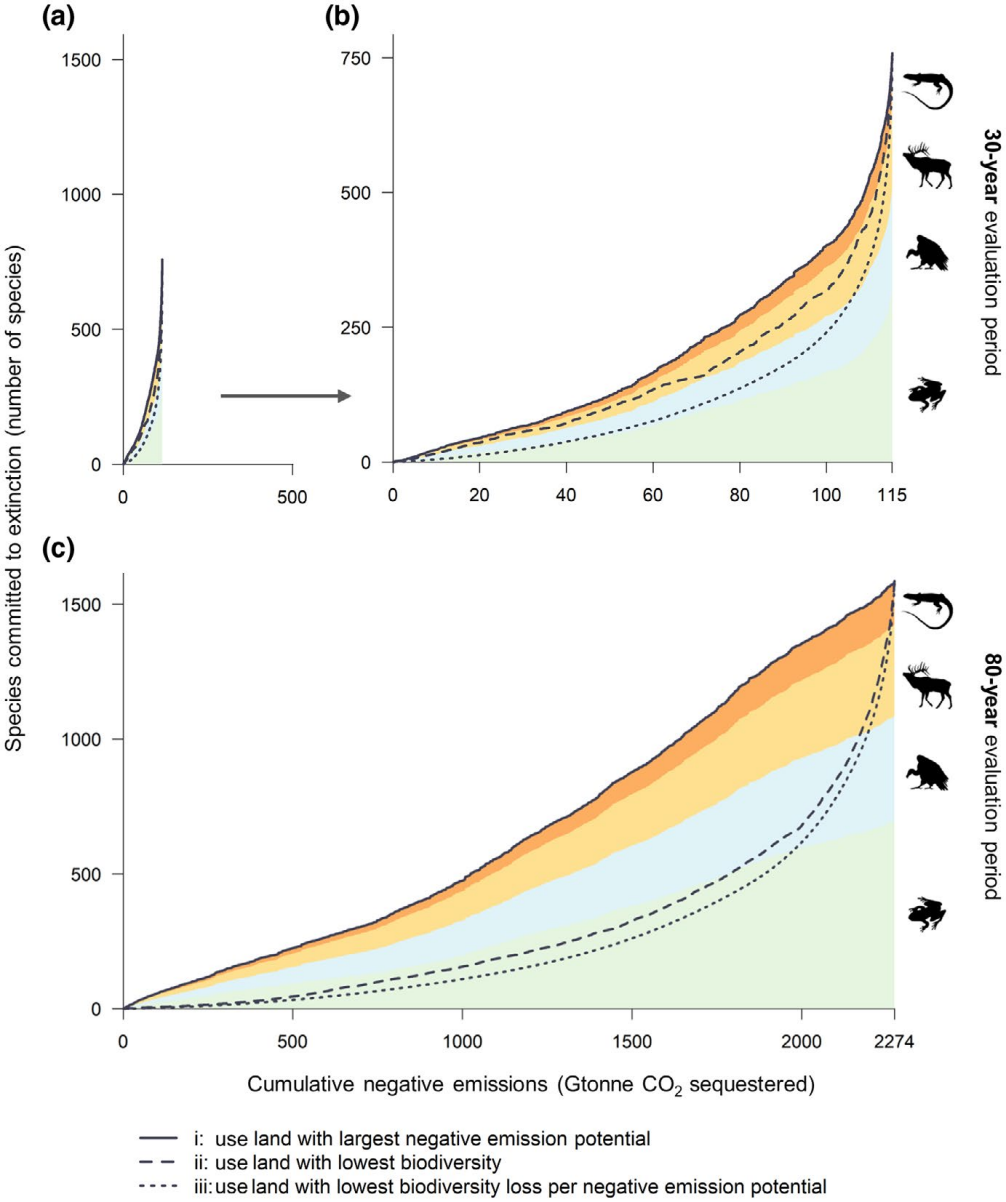
Global biodiversity loss curves for cumulative negative emissions from crop‐based BECCS. The estimated amount of species that becomes committed to extinction due to land‐use change for crop‐based BECCS is shown as a function of cumulative negative emissions. Results are shown for (a) a 30‐year evaluation period, (b) a scaled version of the 30‐year results (note the different axes) and (c) an 80‐year evaluation period. The relation between biodiversity loss and negative emissions differs depending on which land allocation criterion is used (i–iii). Results for the four classes of terrestrial vertebrates, reptiles, mammals, bird and amphibians, are shown using criterion i. Note that maximum cumulative negative emissions over 80 years far exceed negative emission demand in mitigation pathways. BECCS, bioenergy with carbon capture and storage

The biodiversity impacts of local LUC for BECCS can be aggregated into global *biodiversity loss curves* (Figure 2). These curves show the estimated global loss of vertebrate species richness over increasing amounts of cumulative negative emissions achieved with crop‐based BECCS. The large differences between a 30‐ and an 80‐year evaluation period found in Section 3.1 are also reflected in these curves (note the difference in *x*‐axis scaling). In fact, total cumulative negative emissions under 80 years even (far) exceed demand for negative emissions in mitigation pathways. The biodiversity loss curves show that across different classes of vertebrates, amphibians may be most vulnerable, not just in absolute species loss, but also in the share of species lost. For example, 5% of amphibian species would become committed to extinction at maximum sequestration over 30 years, versus 2%–3% for reptiles, mammals and birds. Reaching these maximum levels of negative emissions in these graphs requires conversion of all land included in this analysis (see Section 2.2), which explicitly cannot be part of any realistic scenario. At lower amounts of negative emissions, less land is required and different criteria can be used to select locations for bioenergy crop plantations, resulting in different biodiversity loss curves. We find that minimizing the amount of land used for negative emissions (criterion i), or avoiding the most biodiverse and vulnerable areas (criterion ii) results in more global extinctions per negative emissions than using locations with the lowest species loss per negative emissions potential (criterion iii). As an example, when sequestering 80 Gtonne of CO_2_ over 30 years, minimizing biodiversity loss *per* negative emissions is estimated to result in 137 species committed to extinction (116–164, 95% confidence interval), while minimizing land use doubles that to 272 species (232–321, 95% confidence interval; Figure S5). The geographical patterns of the three land allocation criteria are detailed in Figure S6.

Cumulative negative emissions from crop‐based BECCS in Figure 2c far exceed the requirements of 1.5°C consistent pathways, which are in the order of 100–1000 Gtonne of negative CO_2_ emissions over the 21st century—from BECCS and all other carbon dioxide removal (CDR) options *combined* (Rogelj et al., 2018). While we explicitly do not provide a scenario analysis in this study, the quantities of crop‐based BECCS should be viewed in the light of these CDR requirements and the role of other CDR options (including the use of biomass residues rather than dedicated bioenergy crops for BECCS), meaning *much* less crop‐based BECCS would ever be needed than the maximum theoretical potential values presented here. Expressed per year, overall CDR required in 1.5°C consistent pathways ranges 0–20 Gtonne CO_2_ per year, depending on pathway and decade (Rogelj et al., 2018). In line with this order of magnitude, Table 1 shows the estimates of land requirements and resulting vertebrate species loss at different levels of *annual* negative emissions from crop‐based BECCS. As a reference for the land requirements, global cropland (excluding pastures) covered an estimated 1556 Mha in 2019 (FAOSTAT, 2021). The amounts of electricity generated with crop‐based BECCS at these levels of negative emissions are given in Table S2.

**TABLE 1:** Estimates of land requirements and global biodiversity loss for negative emissions from crop‐based BECCS. Land requirements and terrestrial vertebrate species committed to extinction are estimated for different annual negative emission potentials, and for the three land allocation criteria: (i) use land with largest negative emissions potential, (ii) use land with the lowest biodiversity and (iii) use land with the lowest biodiversity loss per negative emissions potential. Note that (averaged) annual negative emissions larger than 3.8 Gtonne/year are impossible (NA) over a 30‐year evaluation period, as cultivation locations with net negative emissions run out

Land allocation criterion	30‐year evaluation period		80‐year evaluation period	
	Area required (Mha)	Species committed to extinction^a^	Area required (Mha)	Species committed to extinction^a^
	i/ii/iii	i/ii/iii	i/ii/iii	i/ii/iii
Negative emission potential from crop‐based BECCS				
0.5 Gtonne/year	84/295/86	36/26/9	67/171/69	26/3/1
1 Gtonne/year	138/462/184	66/56/24	98/311/153	47/5/2
2 Gtonne/year	309/756/426	166/134/76	155/541/340	82/9/6
5 Gtonne/year	NA	NA	361/1106/817	186/29/22
10 Gtonne/year	NA	NA	678/1705/1319	358/111/73

Abbreviation: BECCS, bioenergy with carbon capture and storage.

^a^ Refers to the global‐equivalent amount of terrestrial vertebrate species becoming committed to extinction.

Our land requirement and species loss estimates in Table 1 are uncertain, as discussed in Section 4, but three key patterns emerge. First, species loss is several times lower under land allocation criterion iii (use land with the lowest species loss per negative emissions) as compared to the other land allocation criteria. This effect is even larger over a longer 80‐year time period and at lower amounts of annual negative emissions, as this allows selecting the most optimal sites. Second, land requirements increase approximately linearly with negative emissions (within the 0–10 Gtonne CO_2_ per year range of Table 1), but species loss follows different patterns, depending on land allocation criterion. When minimizing land use (criterion i), species loss increases approximately linearly with negative emissions. When avoiding the more biodiverse areas (criterion ii) and, in particular, when using ‘optimal’ land allocation (criterion iii), species loss is initially relatively low, but increases sharply with additional negative emissions, as the most optimal sites are quickly depleted and high‐biodiversity and/or low negative emission areas are increasingly required. Third, while over an 80‐year evaluation period less land is required for the same amount of average *annual* negative emissions (as compared to 30 years), this difference is even larger in terms of species loss: around 10 times fewer species are lost per annual amount of negative emissions under the 80‐year evaluation period. In addition to lower land requirements per sequestration, this can be attributed to lands with relatively low biodiversity becoming able to yield negative emissions over the 80‐year evaluation period (Figure S6).

### 3.3 The biodiversity loss trade‐off of crop‐based BECCS: LUC versus mitigated climate change

**FIGURE 3:**
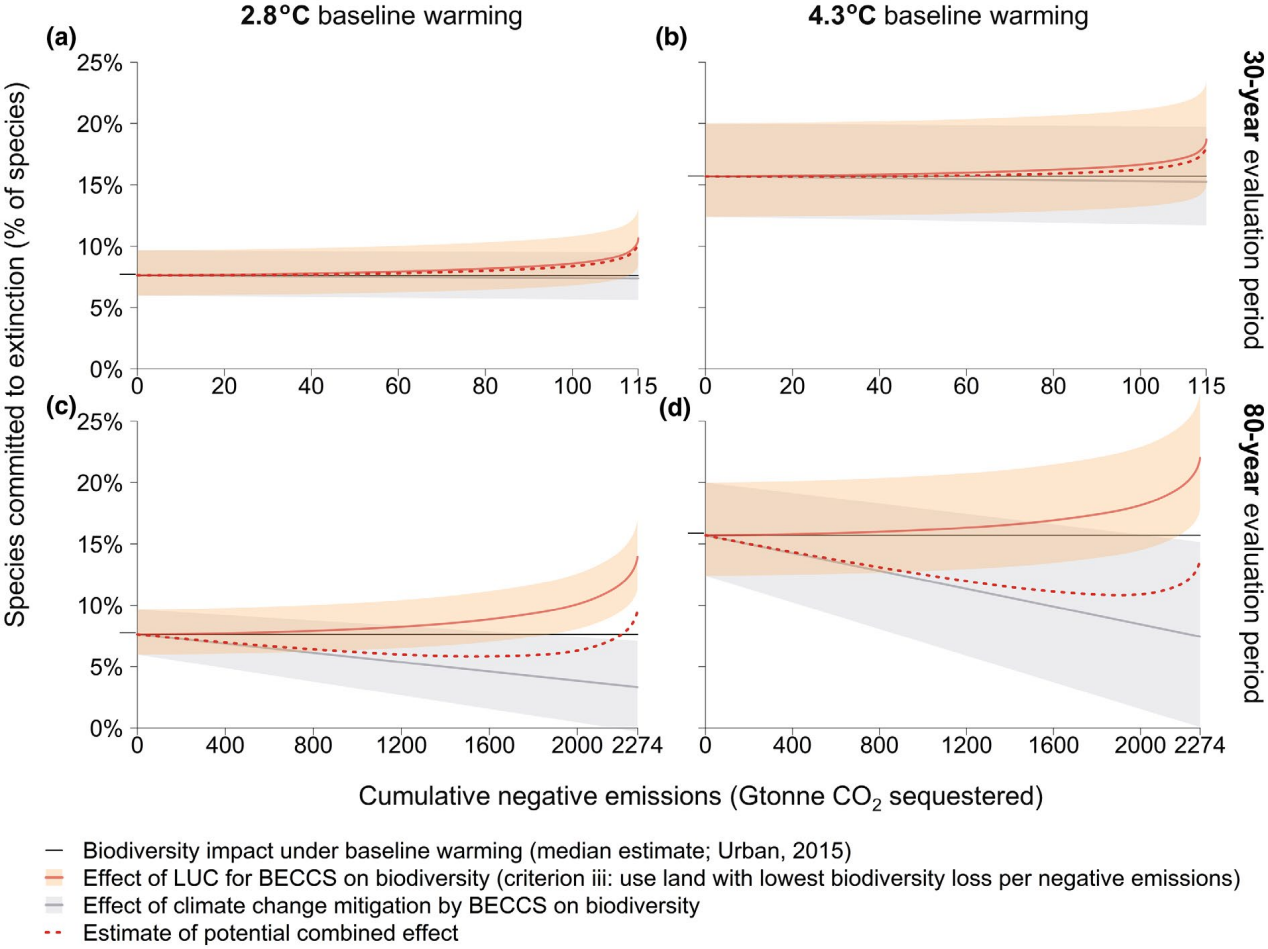
Exploration of the combined effect of LUC *for* BECCS and mitigated climate change *by* BECCS on global terrestrial vertebrate biodiversity. Species committed to extinction is shown as a function of cumulative negative emissions from crop‐based BECCS, over the specified evaluation period. Results are presented for the use of BECCS over 30 and 80 years (panels a, b and c, d respectively; note the different *x*‐axis scaling), and for two baseline warming scenarios, 2.8 and 4.3°C warming by 2100, as compared to pre‐industrial levels (in line with RCP 6 and 8.5; Clarke et al., 2014). The *y*‐axis intercept shows the assumed biodiversity impact of climate change under baseline warming, without BECCS (based on median estimates by Urban, 2015). With increasing negative emissions from BECCS come increasing effects of LUC (red line; criterion iii) and mitigated climate (grey line). Their combined effect is estimated via subtraction (red dotted line), but excludes interaction effects. Shading represents an exploratory estimate of the 2.5th–97.5th percentile uncertainty range, based on the reported uncertainty of the individual effects of LUC and prevented climate change (see Section 2.6). BECCS, bioenergy with carbon capture and storage; LUC, land‐use change

While LUC towards bioenergy crop plantations results in biodiversity loss, climate change mitigation by crop‐BECCS could also help *prevent* further biodiversity loss. Figure 3 shows that the effect of climate change (without any BECCS deployment) could lead to 8%–16% loss of global terrestrial (vertebrate) species in 2.8 and 4.3°C global warming scenarios respectively (based on Urban, 2015). More negative emissions from crop‐based BECCS means increasing effects of LUC on biodiversity (red solid line), but also decreasing effects of climate change (grey line). Their combined effect can be explored by addition (dotted line), though the true interaction is much more complex, as discussed below.

Over a 30‐year evaluation period, biodiversity loss from LUC likely outweighs prevented biodiversity loss from BECCS‐mitigated climate change at all levels of cumulative negative emissions, though both effects are uncertain (Figure 3a,b). Biodiversity loss from LUC is exacerbated under other, less optimal land allocation criteria (e.g. criterion i: minimizing overall land use; Figure S7). At higher cumulative negative emissions, more biodiverse land is required explaining the increase in biodiversity impacts from LUC towards the right side of the graphs. The positive influence of climate change mitigation on biodiversity is small, as negative emissions that can be achieved over 30 years are limited.

Over an 80‐year evaluation period, more negative emissions can be achieved with BECCS per amount of land used. This means the climate change mitigation effect on biodiversity is larger and more warming‐related species extinctions could possibly be averted (Figure 3c,d). When assuming a 2.8°C baseline (i.e. an assumed average global warming of 2.8°C without BECCS), adding the long‐term (80‐year) deployment of crop‐based BECCS is likely to prevent more species loss from climate change than would be lost due to LUC under optimal land allocation (Figure 3c). However, the size of these effects (see shaded areas) and their interaction (not quantified) are highly uncertain, and averting species loss is based on the assumption that negative emissions contribute to 2100 climate targets, which for an 80‐year evaluation period implies that *all* BECCS capacity is in place in 2020 and maintained until (at least) 2100. Under less optimal land allocation criteria (e.g. criterion i: minimizing overall land use), LUC effects likely outweigh climate mitigation effects (Figure S7). The effect of climate change mitigation on biodiversity is non‐linear and strongest when preventing very high temperatures. Therefore, when assuming 4.3°C warming (without the influence of BECCS), the long‐term deployment of crop‐based BECCS could avert more global species loss from climate change (Figure 3d).

## 4 DISCUSSION

### 4.1 The biodiversity impact of LUC for crop‐based BECCS

We find that the land conversion required for lignocellulosic crop production for BECCS strongly impacts biodiversity. Considered over a 30‐year evaluation period, achieving annualized negative emissions of 0.5–5 Gtonne CO_2_ per year requires up to hundreds of Mha of land and commits tens of terrestrial vertebrate species to extinction. These impacts are substantially lower when considering an 80‐year evaluation period to reach these same levels of annualized sequestration, with required cultivation areas changed and reduced in overall size by around 25% and a (resulting) loss of several species. Over both evaluation periods, potential biodiversity loss per unit sequestration is lower at low levels of BECCS deployment, and further reduced when selecting optimal locations (low biodiversity loss per unit sequestration), rather than, for instance, minimizing overall land use. On the other hand, biodiversity loss can increase to well over 10 species committed to extinction for *each* additional Gtonne of CO_2_ sequestered on highly biodiverse locations, such as tropical islands and coastal areas, even when considered over an 80‐year evaluation period. We did not perform an explicit scenario analysis here, but it becomes clear that large‐scale lignocellulosic crop‐based BECCS could commit tens of terrestrial vertebrate species to extinction.

It is important to consider that biodiversity is multifaceted (Pereira et al., 2013). This focused on global species richness and potential extinctions, which puts emphasis on ecoregions with large amounts of endemic species. Including multiple biodiversity indicators has proven relevant in land‐based assessments (Marquardt et al., 2019) and other dimensions of biodiversity that should be included are species abundance and local species richness (Ceballos et al., 2017; Newbold et al., 2015). The vulnerability of these indicators to LUC for bioenergy crop cultivation could be quantified using recently developed impact factors for land use and climate change on local mean species abundance (Schipper et al., 2020), which would allow a more overarching view of this LUC‐driven impact on biodiversity. Such analysis should also look at species groups beyond terrestrial vertebrates that have been shown to be vulnerable to LUC and climate change, importantly plants (Di Marco et al., 2019) and insects (Oliver et al., 2016; van Klink et al., 2020), and could include the wider effects of species loss on ecosystem functioning (Allan et al., 2015). In addition, the potential impacts of bioenergy crop cultivation beyond the use of land should be considered too, including the possible introduction of invasive (bioenergy crop) species (Davis et al., 2010), eutrophication from fertilizers and the toxic effects of pesticide use, in particular on nearby aquatic ecosystems (Immerzeel et al., 2014).

We quantified uncertainty in LUC impacts using the 95% confidence intervals for the global‐equivalent species loss factors by Chaudhary and Brooks (2018), representing statistical uncertainty in the underlying SARs. However, the impacts of large‐scale LUC on *global* vertebrate species richness are inherently difficult to quantify, as empirical data are typically lacking, and not all uncertainties were quantified here. Species loss factors have, for instance, not been specifically derived for the perennial lignocellulosic bioenergy plantations considered in this study (i.e. short‐rotation coppiced trees and *Miscanthus* or switchgrass). In our analysis, we thus used species loss factors (by Chaudhary & Brooks, 2018) for intensively managed plantation forestry, which represent tree monocultures after recent clear‐cut. We also considered loss factors for intensively managed cropland, which lead to (very) similar results, though with a systematically slightly *lower* biodiversity impact (Table S3). The reason being a higher ‘affinity’ (on which species loss factors are based) of the terrestrial vertebrate classes for cropland as compared to plantation forestry, which was determined by Chaudhary and Brooks (2018) using the IUCN Habitat Classification Schemes (2021) and empirical data presented by Newbold et al. (2015). The development of bioenergy crop plantation‐specific species loss factors could improve the estimates of LUC impacts on species richness, though more uncertainty may actually derive from the SARs on which species loss factors are based. For instance, the SARs that underlie the species loss factors used in this study are dependent on a scaling factor ‘*z*’, which is differentiated for islands, forests and non‐forests (Chaudhary & Brooks, 2018). SARs may not always be the best described by such a power law (Storch et al., 2012), but if described this way, *z*‐values could also be further distinguished per biome (Kehoe et al., 2017), resulting in a potentially systematic difference with the present analysis.

### 4.2 The combined biodiversity effects of LUC and climate change mitigation of BECCS

We tentatively explored the trade‐off between species committed to extinction due to LUC for BECCS and the potential species preserved due to BECCS‐mitigated climate change. Over a 30‐year period, LUC effects likely outweigh mitigated climate effects for all warming scenarios and land allocation criteria. This suggests that over shorter evaluation periods, BECCS has a net negative effect on global vertebrate species richness. Over an 80‐year period, our estimate of the combined effects of LUC and preventing climate change suggests that the deployment of crop‐based BECCS likely prevents more species loss from climate change than would be lost due to LUC. This only holds, however, under optimal land allocation. For these 80‐year results in particular, there is the additional consideration that our biodiversity results assume that *all* negative emissions contribute to 2100 climate targets, that is that all BECCS capacity is in place in 2020. When mitigation is achieved later, however, the positive effects of climate change mitigation on biodiversity will be lower. Furthermore, it may not be realistic to maintain this land use for 80 years.

For climate change mitigation, uncertainties were quantified based on the (combined) 95% confidence interval of the species loss meta‐analysis (Urban, 2015) and climate response modelling (Van Vuuren et al., 2020) used in this study. The meta‐analysis concerns all terrestrial species, including insects and plants, and a meta‐regression of climate sensitivity was not explicitly included per taxon. While our use of the aggregated climate sensitivity for the four terrestrial vertebrate classes adds additional uncertainty that we could not quantify, the original meta‐analysis showed no significant differences in extinction risks across taxa (with only a potential, non‐significant, trend for higher extinction risk in amphibians; Urban, 2015), which lends some support to the use of the aggregated sensitivity. The climate sensitivity of global species richness might, however, simply be larger, as suggested by an earlier meta‐analysis (Thomas et al., 2004), meaning mitigating effect of BECCS could preserve more species. A promising alternative to meta‐analysis is the use of process‐based approaches to predict the impacts of climate change on biodiversity (Bouchet et al., 2019; Briscoe et al., 2019; Evans et al., 2016; Yates et al., 2018). The climate change mitigating effect of negative emissions from BECCS includes uncertainty ranges (Van Vuuren et al., 2020), but excludes various factors such as carbon cycle feedbacks and non‐temperature climate effects. Moreover, the effect of *negative* emissions is inherently more uncertain, as no empirical data exist on large‐scale negative emissions. Another important consideration here is that BECCS also yields energy. This means that, at least against the current benchmark of a largely fossil‐fuelled energy supply, the relative benefits to the climate of BECCS may be larger than just the negative emissions (see Hanssen et al., 2020). Reducing the use of fossil fuels may have other, direct benefits to biodiversity too, for instance by reducing accidental spills and ecological disturbance from petroleum extraction (e.g. Dale, Parish, et al., 2015).

The combined effect on biodiversity of LUC and preventing climate change with crop‐based BECCS was explored by comparing two independently modelled effects. This tentative approach ignores the interaction effects between reduced climate change and enhanced habitat loss. A more accurate estimate of the effect of BECCS or other land‐based climate change mitigation measures on biodiversity could be achieved by modelling both LUC and (mitigated) climate change in conjunction, for instance by modelling how they simultaneously affect species distributions (Hof et al., 2018; Visconti et al., 2016). Using such an approach, Hof et al. (2018) showed for bioenergy *without CCS* that LUC impacts outweigh the climate change mitigation effects on global vertebrate species richness. For BECCS, this integrated species distribution‐based trade‐off may have a different outcome, owing to (much) larger climate change mitigation potential of BECCS. The species loss factors used in this study are based on SARs (Chaudhary & Brooks, 2018), which enabled direct translation of land requirements for BECCS to species richness impacts. A third approach could use process‐based models that not only include environmental predictors like climate change, but also biotic interactions, dispersal and physiology, leading to a more mechanistic understanding of biodiversity under the influence of LUC and climate mitigation of crop‐based BECCS.

Regardless of how the biodiversity trade‐off between LUC and mitigated climate change from crop‐based BECCS would unfold, mitigating climate change *without* conversion of natural land would have lower impacts on biodiversity. As outlined in the introduction, BECCS based on lower impact biomass feedstocks can be prioritized, that is BECCS based on biomass wastes and residues (Daioglou et al., 2015; Hanssen et al., 2019; Pour et al., 2018), sustainable forestry (Dale, Kline, et al., 2015; Goh et al., 2020; Hanssen et al., 2020; Lundmark et al., 2016; Peura et al., 2018) or cultivated biomass on marginal or abandoned agricultural lands (Campbell et al., 2008; Gelfand et al., 2013) using biodiverse, local and high‐yielding mixtures of species (Robertson et al., 2017; Tilman et al., 2006). Furthermore, dietary change (i.e. less meat consumption) and improved agricultural practices can reduce land requirements for food provisioning, which could free up land for bioenergy crop production without additional natural land conversion (Van Vuuren et al., 2019). Similarly, sustainable irrigation could also improve biomass yields, both for food provisioning and bioenergy, thus reducing pressure on natural land. The effect of sustainable irrigation on crop‐based BECCS may, however, be limited to an estimated 5%–6% global increase in potential negative emissions (Ai et al., 2021). Alongside low‐impact BECCS, other CDR technologies (Fuss et al., 2018; Smith et al., 2016) and more rapid deployment of renewable energy sources (e.g. Van Vuuren et al., 2018) can provide climate change mitigation, although they too have some impact on biodiversity (e.g. Holland et al., 2019; Popescu et al., 2020). In this light, the restoration of ecosystems may contribute to climate change mitigation (Griscom et al., 2017, 2020; Roe et al., 2021) while providing direct benefits to biodiversity, with one prominent option being the restoration of natural forests (e.g. Lewis et al., 2019).

## 5 CONCLUSIONS

Based on this study, we come to the following conclusions:

- LUC for lignocellulosic crop‐based BECCS can lead to global extinctions of vertebrate species. Depending on the BECCS evaluation period and land allocation criteria, and assuming agricultural land used for food provisioning is off limits, sequestering 0.5–5 Gtonne of CO_2_ per year with lignocellulosic crop‐based BECCS could require the conversion of hundreds of Mha of land and commit tens of terrestrial vertebrate species to extinction.
- The evaluation period of a BECCS system is a key factor in determining its biodiversity impact. Per negative emissions achieved, less land is needed and fewer species are committed to extinction due to LUC when BECCS systems are operated longer. The short‐term operation of crop‐based BECCS should always be avoided.
- Biodiversity loss curves for lignocellulosic crop‐based BECCS make clear that to achieve a certain amount of sequestration, minimizing overall land‐use or prioritizing land with the lowest biodiversity results in substantially larger biodiversity impacts than prioritizing optimal locations (lowest biodiversity loss per negative emission potential). This effect is strongest, and relative biodiversity impacts are lowest, at low overall levels of crop‐based BECCS deployment.
- Tentative comparison shows that LUC impacts on global terrestrial species richness may outweigh the positive effects of climate change mitigation, for crop‐based BECCS considered over a 30‐year period. Conversely, for BECCS considered over 80 years, the positive effects of climate change mitigation on biodiversity may (under optimal land allocation) outweigh the negative effects of LUC. However, both effects *and* their interaction are highly uncertain and require further understanding, along with the analysis of additional species groups and biodiversity metrics.
- Factoring in biodiversity means that lignocellulosic crop‐based BECCS should be: (i) deployed as early as possible to allow maximum sequestration before future climate targets, thereby reducing land requirements per negative emissions, (ii) based on biomass grown on optimal cultivation locations (lowest biodiversity loss per negative emission potential) and, most importantly, (iii) used as little as possible where conversion of natural land is involved, looking instead to sustainably grown or residual biomass feedstocks and alternative strategies for CDR.

## CONFLICT OF INTEREST

The authors declare no conflict of interest.

## Supporting information

Supplementary Material

## Data Availability

The data that support the findings of this study are openly available in the DANS EASY archive at https://doi.org/10.17026/dans‐zwh‐x29p.
