# Supplementary material for: Global implications of crop‐based bioenergy with carbon capture and storage for terrestrial vertebrate biodiversity
Source: Glob Change Biol Bioenergy. 2021 Dec 20;14(3):307–21. doi: 10.1111/gcbb.12911 (PMC9299942; doi:10.1111/gcbb.12911)
Supplement: Supplementary file 1 — Supplementary Material [file GCBB-14-307-s001.pdf]

Global implications of crop-based bioenergy with carbon capture and storage for terrestrial vertebrate biodiversity

Supplementary Information

S.V. Hanssen*‡[1], Z.J.N. Steinmann*[2,1], V. Daioglou[3,4], M. Čengić[1], D.P. Van Vuuren[3,4], M.A.J. Huijbregts[1]

* Equal contribution

‡ Corresponding Author

[1]Department of Environmental Science, Institute for Water and Wetland Research, Radboud University, P.O. Box 9010, 6500 GL Nijmegen

[2]Environmental Systems Analysis Group, Wageningen University & Research, Droevendaalsesteeg 3, 6708PB, Wageningen, The Netherlands

[3]PBL Netherlands Environmental Assessment Agency, P.O. box 30314, 2500 GH The Hague, The Netherlands

[4]Copernicus Institute of Sustainable Development, Utrecht University, Princetonlaan 8a, 3584 CB Utrecht, The Netherlands

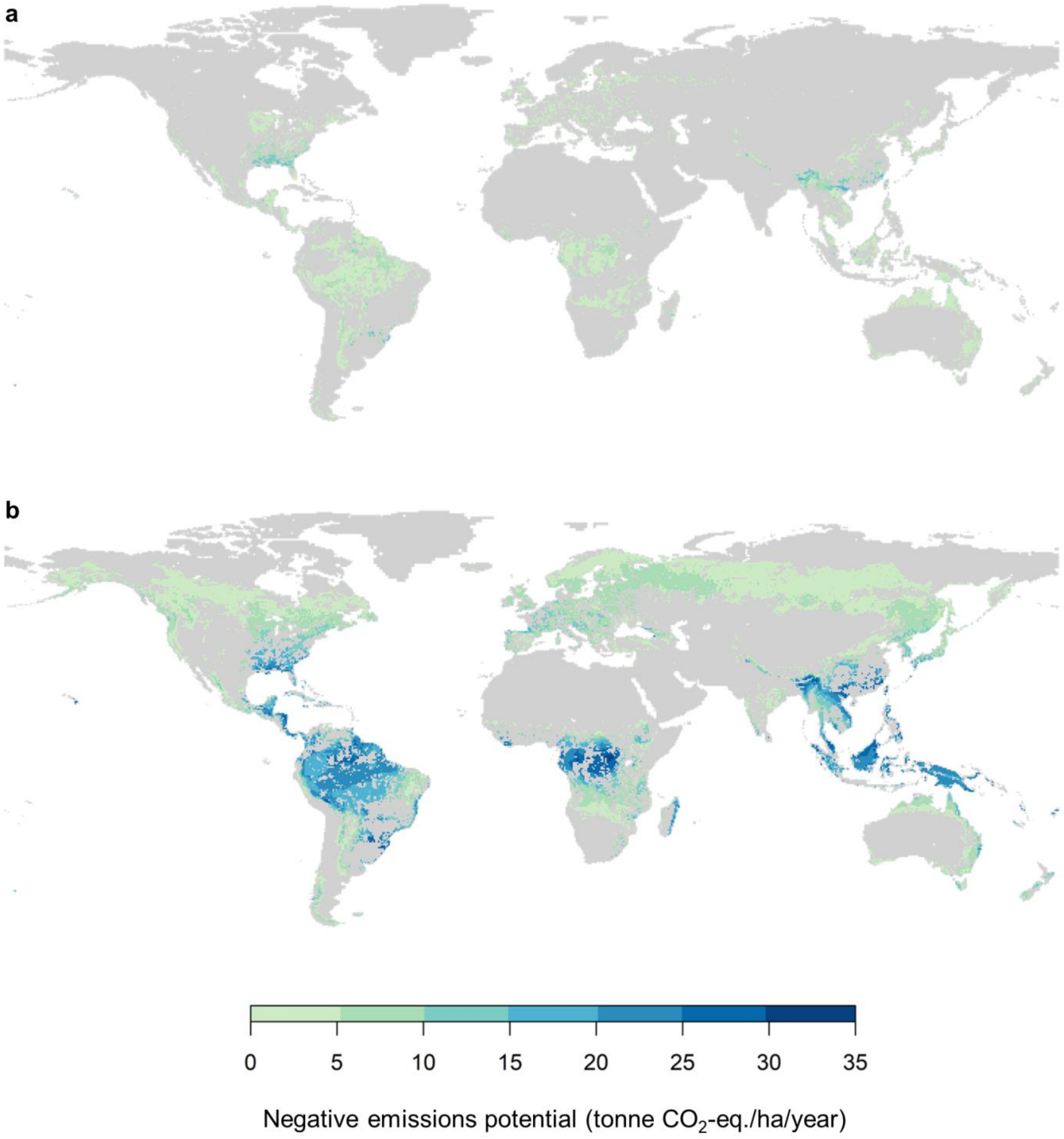

Figure S1 | Net negative emission potential (kg CO2-eq. / ha / year) of BECCS electricity. Negative emission potential is shown (**a**) over a 30 year evaluation time, and (**b**) over an 80 year evaluation time, and is based on Hanssen et al., (2020). It is assumed that 80% of stem biomass in the original vegetation is used for BECCS.

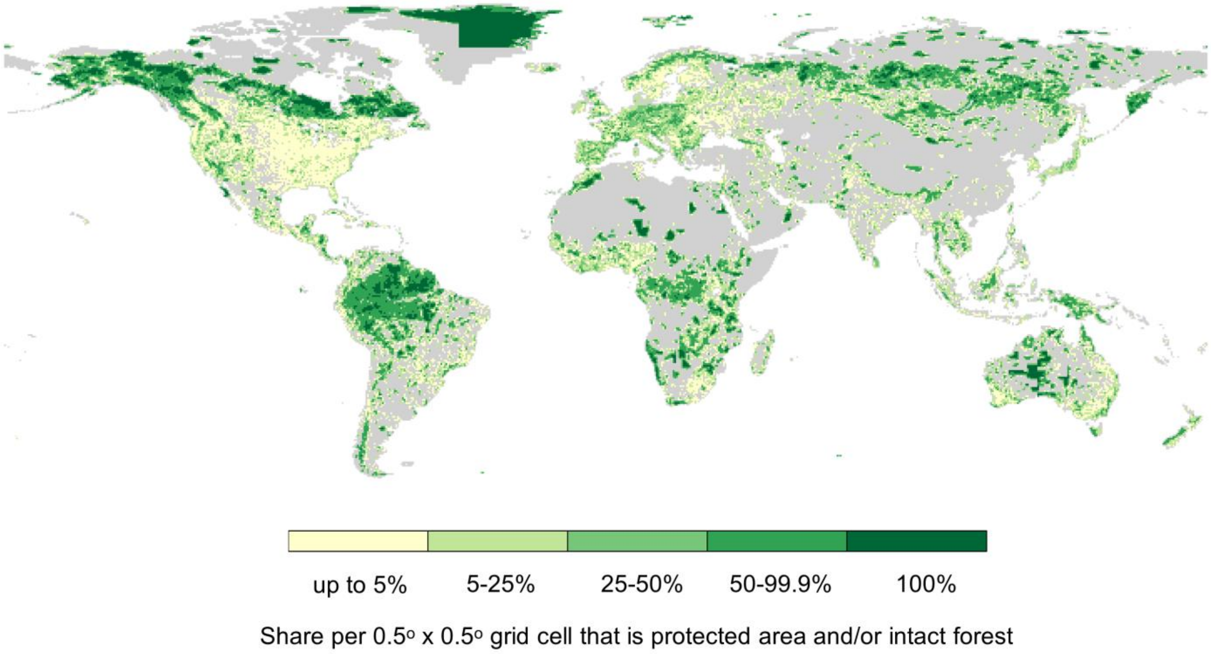

Figure S2 | Share of protected area and/or intact forest per 0.5º x 0.5º grid cell. Currently protected areas were based on UN WCMC (2019). So-called intact forests were based on Potapov et al. (2017) and are defined as natural areas (including non-forest ecosystems) without human activities that are larger than 50 km^2 and at least 10 km at their broadest point; they are thought to be large enough to maintain all native biodiversity. All protected areas and intact forests were excluded from our analysis. When entire cells are covered by protected areas and/or intact forests, they were altogether excluded, when part of a cell is covered, that *share* of the cell was excluded from our analysis (i.e., from modelled negative emissions of BECCS and associated biodiversity loss estimates).

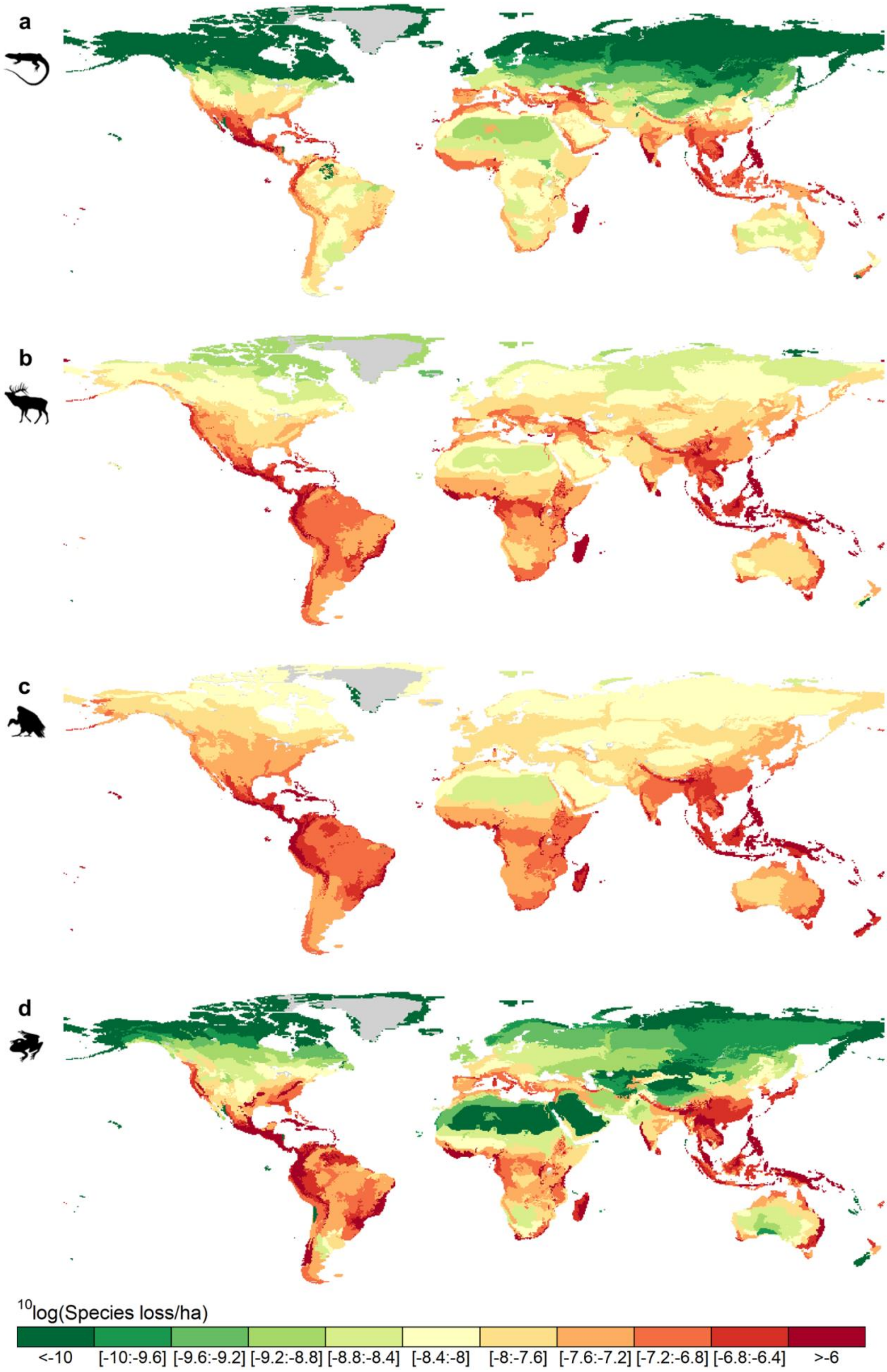

Figure S3 | Global-equivalent potential species loss factors for the conversion to intensive plantation forestry. Intensive plantation forestry represents bioenergy crop plantations in our study. Loss factors were determined by Chaudhary & Brooks, 2018. They are shown for: (**a**) reptiles, (**b**) mammals, (**c**) birds, and (**d**) amphibians. Note that a log scale is used.

Table S1 | Estimated terrestrial biodiversity loss due to global warming. Biodiversity loss refers to species committed to global extinction. All values are based on Urban (2015). We looked at two scenarios for global temperature increase: 2.8 °C and 4.3 °C, representing the approximate amount of warming expected by 2100 as compared to pre-industrial temperatures in representative concentration pathways (RCPs) 6.0 and 8.5, respectively (Clarke et al., 2014). The 95% confidence interval (as shown by Urban, 2015) is indicated in brackets. Note that a temperature interval of 1 °C was used in estimating biodiversity loss due to warming over an 80 year evaluation period, corresponding to the approximate temperature reduction BECCS could achieve with maximum cumulative negative emissions over this evaluation period. For the 30 year evaluation period an interval of 0.2 °C was used.

**Scenario**	**Temperature interval[1]**		**Biodiversity loss[2] due to warming**	**Biodiversity loss[2] prevented by mitigating warming**
	°C		% of species	% of species / °C
30 year evaluation period	from	to	def. *(2.5 - 97.5th %)*	def. *(2.5 - 97.5th %)*
2.8 °C biodiversity impact	2.6	2.8	0.7 *(0.6 - 0.9)*	3.5 *(3.2 - 4.4)*
4.3 °C biodiversity impact	4.1	4.3	1.3 *(1.1 - 1.8)*	6.4 *(5.4 - 8.9)*
80 year evaluation period				
2.8 °C biodiversity impact	1.8	2.8	3.1 *(2.5 – 3.7)*	3.1 *(2.5 – 3.7)*
4.3 °C biodiversity impact	3.3	4.3	5.8 *(4.7 – 7.5)*	5.8 *(4.7 – 7.5)*

Abbreviations: def. = default. **Notes:** [1] as compared to pre-industrial temperatures; [2] terrestrial biodiversity.

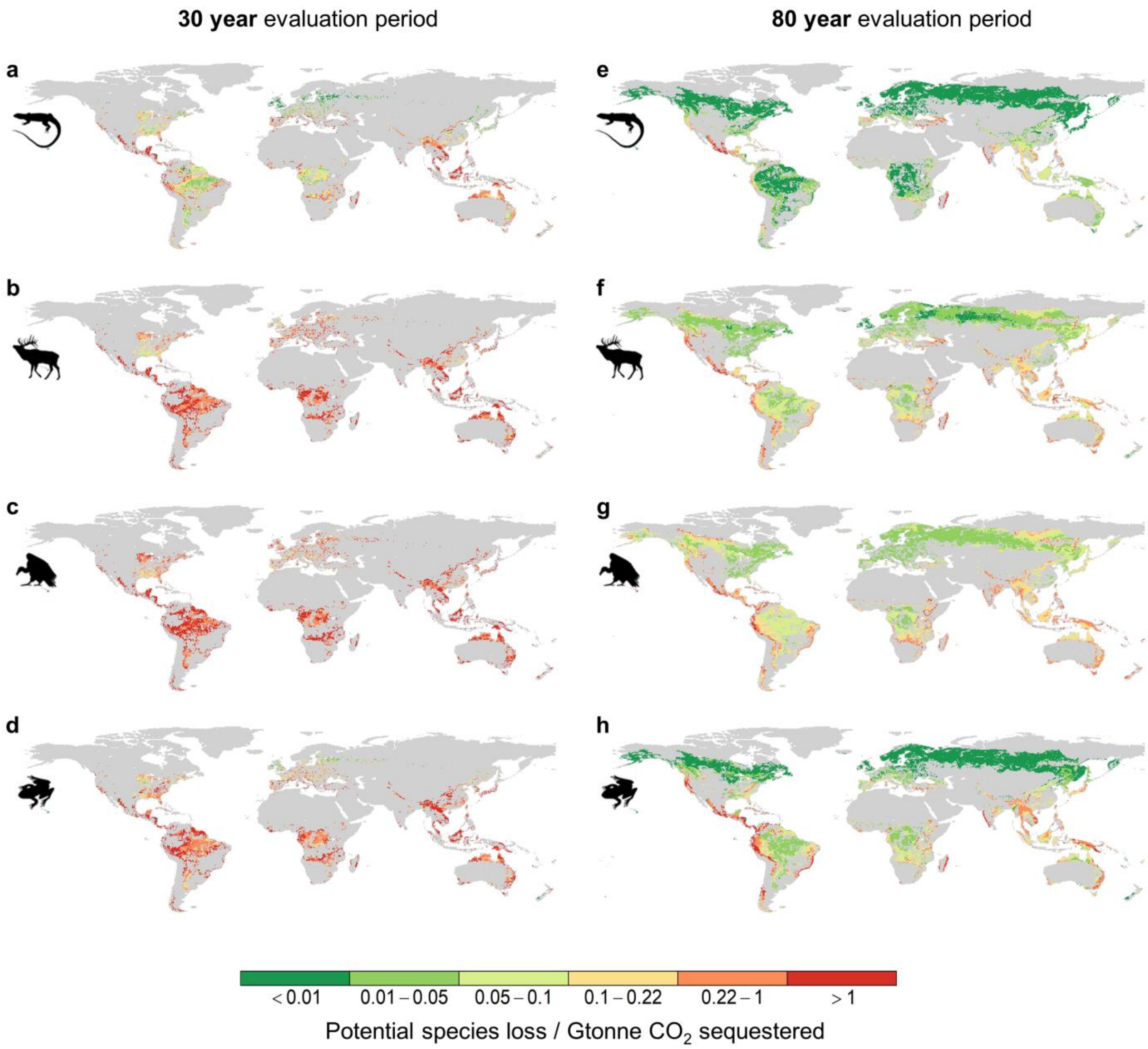

Figure S4 | Maps of global species loss due to negative emissions from crop-based BECCS for four classes of terrestrial vertebrates. Indicated are the potential number of species that become committed to global extinction due to LUC, expressed per Gigatonne of CO_2 sequestered with BECCS over a 30 year evaluation period, for: (**a**) reptiles, (**b**) mammals, (**c**) birds, and (**d**) amphibians. Results for an 80 year evaluation period are shown in panels (**e-h**). Grey areas were excluded from our analysis and comprise: agricultural land (cropland and pasture), urban areas, inland waters, protected areas, intact forests, areas with low bioenergy crop yields (<5% of global maximum yields) and areas that do not achieve net CO_2 sequestration over the time period considered. Grid cells (0.5^{o} x 0.5^{o}) that are partially protected areas or intact forests are fully plotted here, but the protected/intact share is excluded from the remaining analysis. *Note that the legend scale differs from Figure 1 in the main text.*

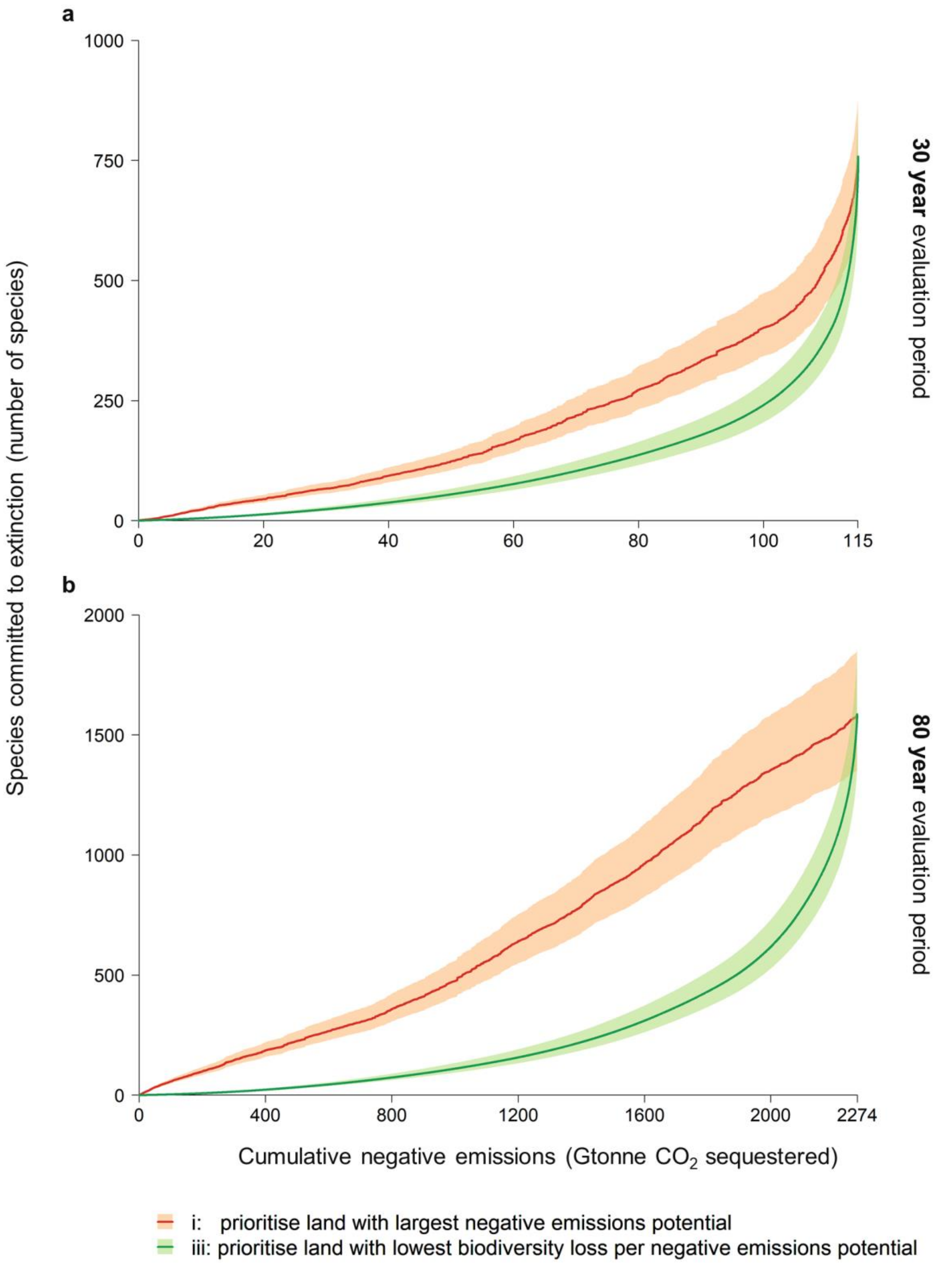

Figure S5 | Estimated uncertainty in global terrestrial vertebrate species loss due to land-use change for BECCS *(based on Chaudhary & Brooks, 2018)***.** The amount of species that become committed to extinction is shown as a function of cumulative negative emissions from crop-based BECCS. The shaded area represents the 2.5 to 97.5th percentile uncertainty range for the impacts of land-use change on biodiversity, as determined by Chaudhary & Brooks (2018), considered for all ecoregions simultaneously. Results are presented for (**a**) a 30 year evaluation period and (**b**) an 80 year evaluation period. The relation between biodiversity loss and negative emissions differs depending on which land allocation criterion (i or iii) is used.

Geographical patterns resulting from different land allocation criteria

What type of areas are prioritised in the global biodiversity loss curves (Figure 2) to achieve a certain amount of cumulative negative emissions from crop-based BECCS, differs for the three different land allocation criteria (Figure S6). When applying criterion i (minimise land-use) the areas in the US South-East, southern parts of South America, small parts of South-East Asia, and eastern parts of Australia are used first. For criterion ii (prioritise least biodiverse lands), almost all available land in Europe and a large portion of land in the Americas and Africa is converted to prevent land conversions in the biodiversity richest areas. With criterion iii (minimise biodiversity loss per negative emissions potential) spatial patterns are in between those of the first two criteria: warm and sub-tropical areas with large negative emission potential are used earlier on, along with European areas with relatively low biodiversity loss. These patterns remain fairly similar over different evaluation periods, except that over a longer evaluation period more areas could yield negative emissions and are therefore included in the analysis.

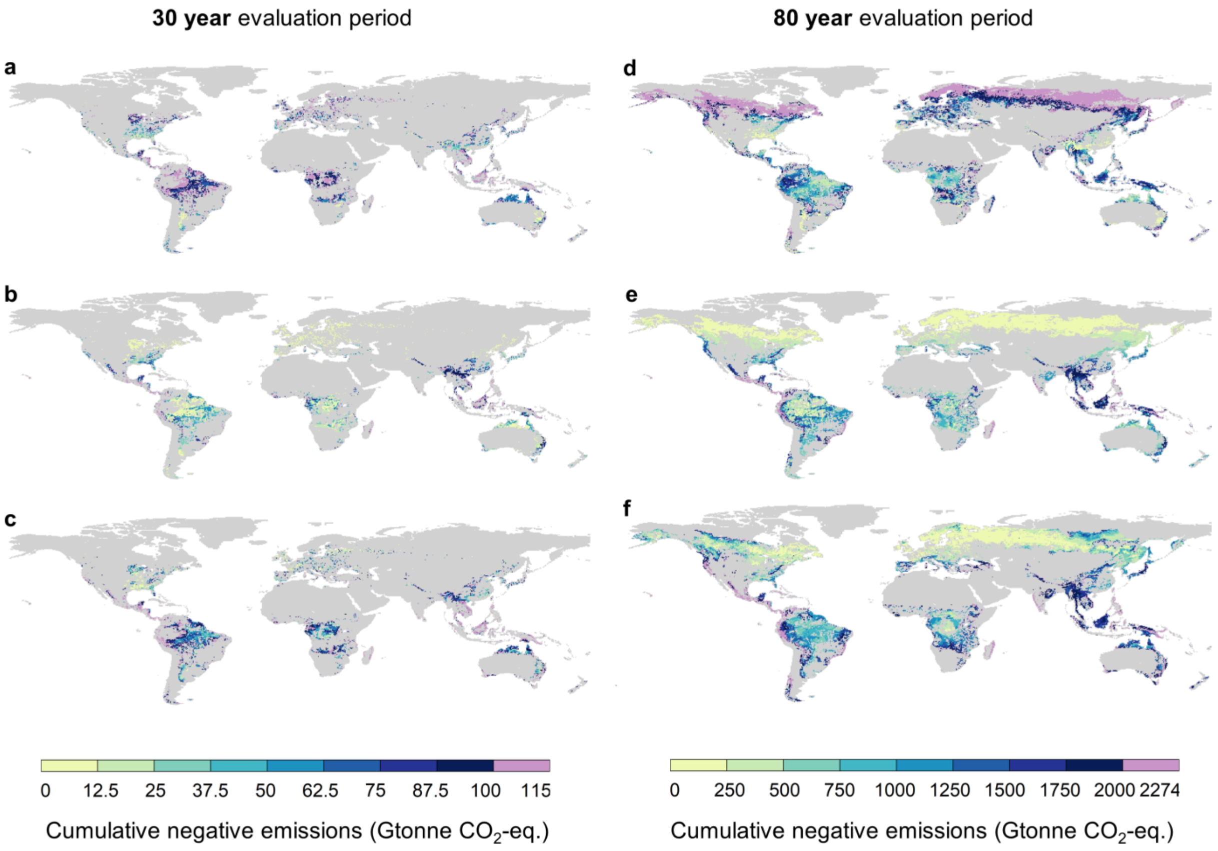

Figure S6 | Locations required to achieve cumulative negative emissions via BECCS under different land allocation criteria. Maps on the left display land areas that would be required for BECCS to achieve a certain level of cumulative negative emissions over a 30 year evaluation period, when (**a**) prioritising land with the largest negative emissions potential (criterion i), (**b**) prioritising land with lowest biodiversity (criterion ii), and (**c**) prioritising land with lowest biodiversity loss per negative emission potential (criterion iii). Maps on the right (**d-f**) display land areas required to achieve a certain amount of cumulative negative emissions over an 80 year evaluation period, under these same three criteria (i-iii, respectively).

Table S2 | Electricity generation from lignocellulosic crop-based BECCS across different potential negative emission levels and land allocation criteria. Electricity generation from lignocellulosic crop-based BECCS is estimated for two evaluation periods, different (global) annual negative emission potentials, and the three land allocation criteria used in this study: i) use land with largest negative emissions potential, ii) use land with lowest biodiversity, and iii) use land with lowest biodiversity loss per negative emissions potential. Note that (averaged) annual negative emissions larger than 3.8 Gtonne/year and associated electricity generation are impossible (NA) over a 30 year evaluation period, as cultivation locations with net negative emissions run out. Part of the reason that annual electricity generation differs between 30 and 80 year evaluation periods is a difference in cultivation areas that are required to achieve the same (average) annual negative emissions potentials. Also note that the levels of electricity generation differ from the default numbers presented by Hanssen et al. (2020). That study assumes burning of initial vegetation, whereas the current study assumes 80% of stem biomass in initial vegetation is used to produce additional electricity (see methods), and also uses different land allocation criteria.

	30 year evaluation period	*80 year evaluation period*
	**Electricity generation** (EJ / year)	**Electricity generation** (EJ / year)
land allocation criterion	i / ii / iii	i / ii / iii
Negative emission potential from crop-based BECCS		
0.5 Gtonne / year	2.4 / 10.0 / 4.1	4.5 / 11.1 / 7.2
1 Gtonne / year	5.4 / 18.7 / 9.1	9.3 / 21.1 / 15
2 Gtonne / year	13.6 / 36.7 / 22.2	19.3 / 38.7 / 31.6
5 Gtonne / year	NA	51.8 / 86.7 / 77.4
10 Gtonne / year	NA	110.4 / 152.3 / 143.8

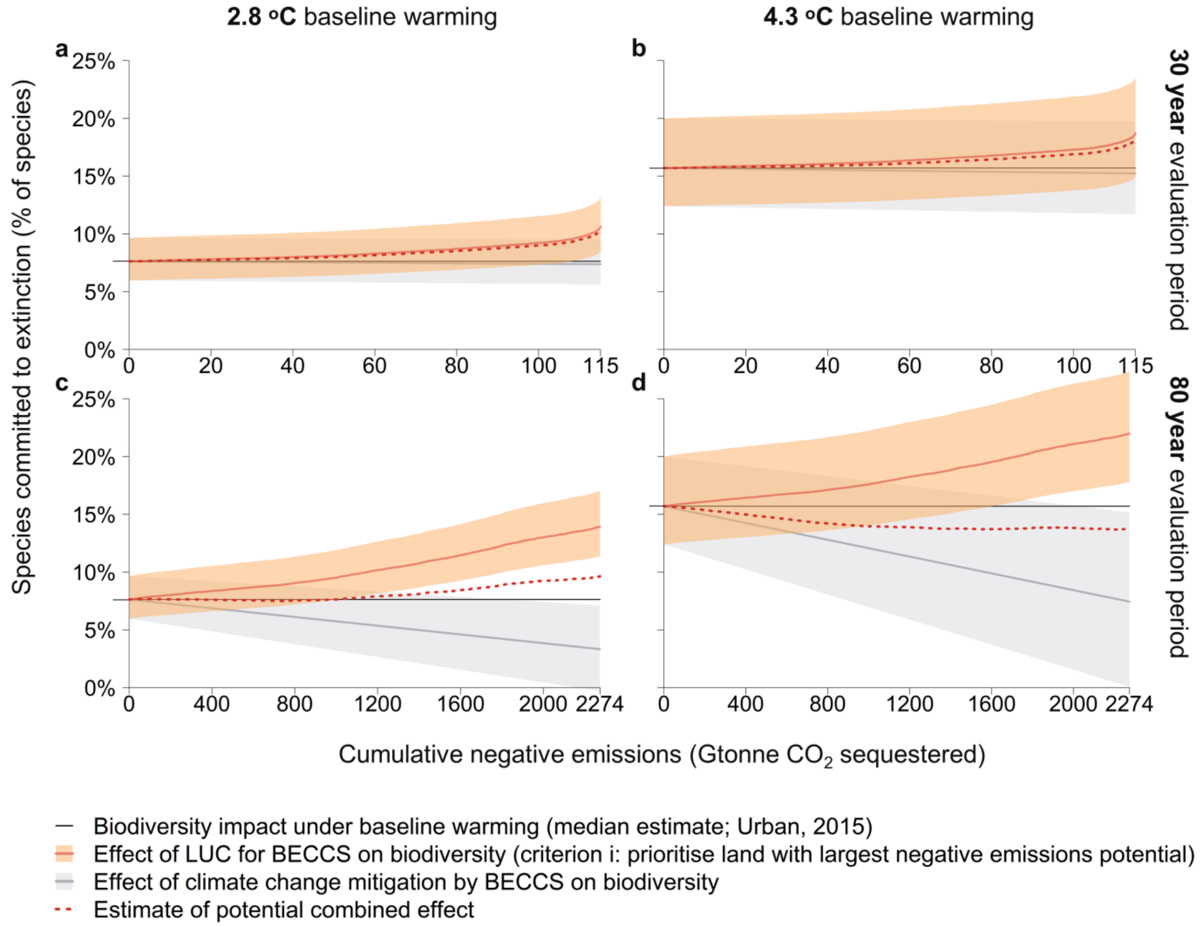

Figure S7 | Exploration of the combined effect of LUC *for* BECCS and mitigated climate change *by* BECCS on global terrestrial vertebrate biodiversity, prioritising land with largest negative emission potential. The amount of species committed to extinction is shown as a function of cumulative negative emissions from crop-based BECCS, over the specified evaluation period. Results are presented for the use of BECCS over 30 and 80 years (panel **a-b** and **c-d**, respectively; note the different x-axis scaling), and for two baseline warming scenarios: 2.8 °C and 4.3 °C warming by 2100, as compared to pre-industrial levels (in line with RCP 6 and 8.5; Clarke et al., 2014). The y-axis intercept shows the assumed biodiversity impact of climate change under baseline warming, without BECCS (based on median estimates by Urban [2015]). With increasing negative emissions from BECCS come increasing effects of land-use change (red line; criterion i) and mitigated climate (grey line). Their combined effect is estimated via subtraction (red dotted line), but excludes interaction effects. Shading represents an exploratory estimate of the 2.5 to 97.5th percentile uncertainty range, based on the reported uncertainty of the individual effects of LUC and prevented climate change (see section 2.6), but for instance excludes the uncertainty of their interaction effect.

Table S3 | Estimates of land requirements and global biodiversity loss for negative emissions from crop-based BECCS – specifically using species loss factors for intensively managed cropland instead of intensively managed plantation forestry. Land requirements and terrestrial vertebrate species committed to extinction are estimated for different annual negative emission potentials, and for the three land allocation criteria: i) use land with largest negative emissions potential, ii) use land with lowest biodiversity, and iii) use land with lowest biodiversity loss per negative emissions potential. Note that (averaged) annual negative emissions larger than 3.8 Gtonne/year are impossible (NA) over a 30 year evaluation period, as cultivation locations with net negative emissions run out.

	30 year evaluation period		*80 year evaluation period*	
	**Area required** (Mha)	**Species committed to extinction**[1]	**Area required** (Mha)	**Species committed to extinction**[1]
land allocation criterion	i / ii / iii	i / ii / iii	i / ii / iii	i / ii / iii
Negative emission potential from crop-based BECCS				
0.5 Gtonne / year	84 / 297 / 93	34 / 24 / 8	67 / 165 / 67	25 / 2 / 1
1 Gtonne / year	138 / 448 / 187	62 / 51 / 22	98 / 291 / 148	44 / 4 / 2
2 Gtonne / year	309 / 752 / 426	156 / 128 / 72	155 / 531 / 334	77 / 7 / 5
5 Gtonne / year	NA	NA	361 / 1106 / 829	176 / 26 / 20
10 Gtonne / year	NA	NA	678 / 1701 / 1334	340 / 104 / 68

[1] Refers to the global-equivalent amount of terrestrial vertebrate species becoming committed to extinction.

References

Chaudhary, A. & Brooks, T.M. (2018) Land use intensity-specific global characterization factors to assess product biodiversity footprints. *Environmental Science and Technology* 52, 5094-5104.

Clarke, L. et al. (2014) Assessing Transformation Pathways. In: Climate Change 2014: Mitigation of Climate Change. IPCC Fifth Assessment Report [Edenhofer O, et al. (eds.)]. Cambridge University Press, Cambridge, UK and New York, NY, USA.

Hanssen, S.V., Daioglou, V., Steinmann, Z.J.N., Doelman, J., van Vuuren, D.P. & Huijbregts, M.A.J. (2020) The climate change mitigation potential of bioenergy with carbon capture and storage. *Nature Climate Change* 10, 1023-1029.

Potapov, P. et al. (2017) The last frontiers of wilderness: tracking loss of intact forest landscapes from 2000 to 2013. *Science Advances* 3, e1600821.

van Vuuren, D.P., van der Wijst, K.-J., Marsman, S., van den Berg, M., Hof, A.F. & Jones, C.D. (2020) The costs of achieving climate targets and the sources of uncertainty. *Nature Climate Change* 10, 329-334.

UNEP WCMC (2020) World Database on Protected Areas. [Available online at: https://www.protectedplanet.net; accessed September 2019] UNEP-WCMC, Cambridge, UK (2020).

Urban, M.C. (2015) Accelerating extinction risk from climate change. *Science,* 348, 571-573.
